# rs7041 and rs4588 Polymorphisms in Vitamin D Binding Protein Gene (VDBP) and the Risk of Diseases

**DOI:** 10.3390/ijms23020933

**Published:** 2022-01-15

**Authors:** Dominika Rozmus, Janusz Płomiński, Klaudia Augustyn, Anna Cieślińska

**Affiliations:** 1 Faculty of Biology and Biotechnology, University of Warmia and Mazury, 10-719 Olsztyn, Poland; anna.cieslinska@uwm.edu.pl; 2 Clinical Department of Trauma-Orthopedic Surgery and Spine Surgery of the Provincial Specialist Hospital in Olsztyn, 10-561 Olsztyn, Poland; plominsky@poczta.onet.pl; 3 Department and Clinic of Orthopaedics and Traumatology, Collegium Medicum, University of Warmia and Mazury, 10-719 Olsztyn, Poland; 4 Faculty of Medicine, Collegium Medicum, University of Warmia and Mazury, 10-082 Olsztyn, Poland; klaudia.augustyn@student.uwm.edu.pl

**Keywords:** VDBP, vitamin D binding protein, rs7041, rs4588, bone density, diabetes, obesity, COPD, pulmonary tuberculosis, SNP, MD, PD

## Abstract

The purpose of the study was to investigate the role of vitamin D binding protein (VDBP, DBP) and its polymorphism in the vitamin D pathway and human health. This narrative review shows the latest literature on the most popular diseases that have previously been linked to VDBP. Vitamin D plays a crucial role in human metabolism, controlling phosphorus and calcium homeostasis. Vitamin D binding protein bonds vitamin D and its metabolites and transports them to target tissues. The most common polymorphisms in the VDBP gene are rs4588 and rs7041, which are located in exon 11 in domain III of the VDBP gene. rs4588 and rs7041 may be correlated with differences not only in vitamin D status in serum but also with vitamin D metabolites. This review supports the role of single nucleotide polymorphisms (SNPs) in the VDBP gene and presents the latest data showing correlations between VDBP variants with important human diseases such as obesity, diabetes mellitus, tuberculosis, chronic obstructive pulmonary disease, and others. In this review, we aim to systematize the knowledge regarding the occurrence of diseases and their relationship with vitamin D deficiencies, which may be caused by polymorphisms in the VDBP gene. Further research is required on the possible influence of SNPs, modifications in the structure of the binding protein, and their influence on the organism. It is also important to mention that most studies do not have a specific time of year to measure accurate vitamin D metabolite levels, which can be misleading in conclusions due to the seasonal nature of vitamin D.

## 1. Introduction

Recent data suggest that vitamin D deficiency is widespread across Europe. Analysis of 14 population studies revealed that 13% of the 55,844 European individuals had average yearly serum 25(OH)D concentrations <30 nmol/L, regardless of age group, ethnic mix, and the latitude of study populations [1]. According to the US Endocrine Society definition of vitamin D deficiency (<50 nmol/L), the prevalence was 40.4% and dark-skinned ethnic subgroups were more likely to be vitamin D deficient [2]. Due to the darker skin color, melanin blocks the UVB radiation, which is necessary for vitamin D synthesis. Hypovitaminosis D was highly prevalent among pregnant Bangladeshi women, and parity and gestational age were common risk factors of vitamin D deficiency [3].

1,25(OH)_2_D is considered to be the most powerful physiological agent. It stimulates the active transport of calcium, phosphorus, and magnesium. Disorders in vitamin D action can lead to a decrease in the net flux of minerals to the extracellular compartment, which can lead to hypocalcemia and secondary hyperparathyroidism [4]. In addition, low concentrations of calcium and phosphorus will lead to defective mineralization of the bone matrix and rickets [5,6]. Vitamin D is also a regulator of the immune system, where the expression of CYP27B1 in macrophages leads to local production of 1,25-dihydroxyvitamin D ((1,25[OH]_2_D)), which induces the expression of genes encoding antimicrobial peptides [7]. (1,25[OH]_2_D) induces and stimulates autophagy resulting in enhanced bacterial killing, suppresses production of pro-inflammatory cytokines, and prevents overstimulated immune response [8].

The purpose of the study is to investigate and systematize the current knowledge regarding the impact of VDBP polymorphisms on the risk of incidence of various diseases and human health. The “Vitamin D” (calciferol) term refers to two secosteroids: vitamin D2 (ergocalciferol) and vitamin D3 (cholecalciferol). They are both produced from sterol precursors with light in the UVB spectrum of 280 to 320 nm. In fungi and plants, ergosterol is the vitamins’ D2 precursor, while the vitamins’ D3 precursor is 7-dehydrocholesterol (7-DHC), and its high concentration was found in the skin [9]. Vitamin D2 and D3 differ in side chains, but both are converted to 25-hydroxyvitamin D 25(OH)D and 1,25-dihydroxyvitamin D [9,10]. 25OHD is considered to be the best reflection of the vitamin D level in serum [10]. Vitamin D3 can be synthesized endogenously under ultraviolet (UV) light [11].

**Figure 1:**
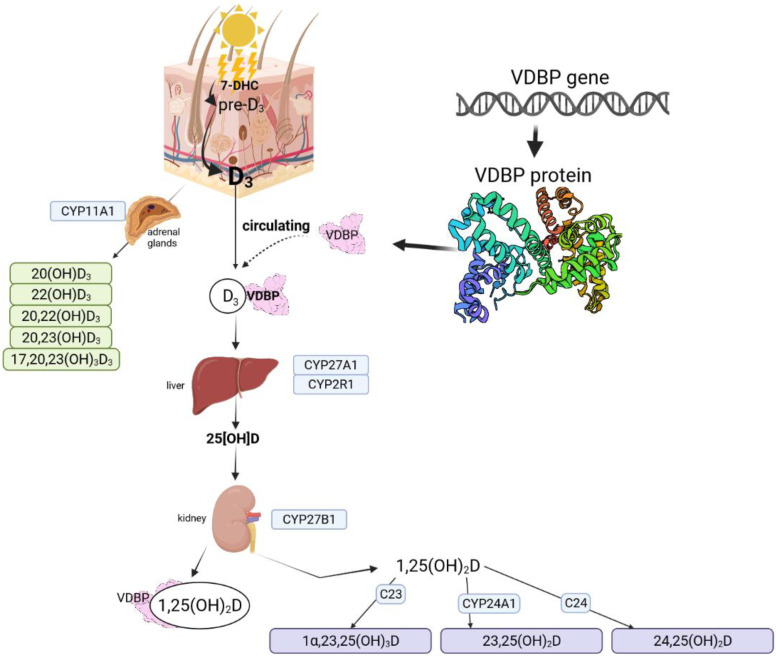
Vitamin D metabolic pathways based on Bikle (2014), Slominski et al. (2012, 2015, 2020), Rozmus et al. (2020) [12,15,16,17,18]. Figure fully created with biorender.com (accessed on 1 November 2021).

Vitamin D photoproduction starts with 7-dehydrocholesterol (7-DHC), which is synthesized and built into the membranes of the epidermis and dermis [12]. During sunlight exposure, the epidermal 7-DHC is converted into pre-vitamin D3 [12,13]. Pre-D3 is thermo-isomerized to the vitamin D3 in the cell membrane. The produced cholecalciferol is removed into extracellular space and reaches the skin’s capillary by diffusion [12]. Prolonged exposure to solar ultraviolet radiation while pre-vitamin D3 synthesis reaches a plateau of 10 to 15 percent of the original 7-DHC has an increasing effect only on lumisterol and tachysterol, two biologically inactive photoisomers [13]. Lumisterol can revert back to pre-D3 in the dark, but maximum levels of pre-D3 lead to the accumulation of inactive luminsterol with continued UV exposure. The production of lumisterol and tachysterol has a protective effect against the production of toxic amounts of D3 [9]. During activation and inactivation processes, cytochrome P450 (CYP) enzymes are involved throughout the vitamin D3 pathway [14,15]. The pathway is presented in Figure 1 (based on [12,15,16,17,18].

**Figure 2:**
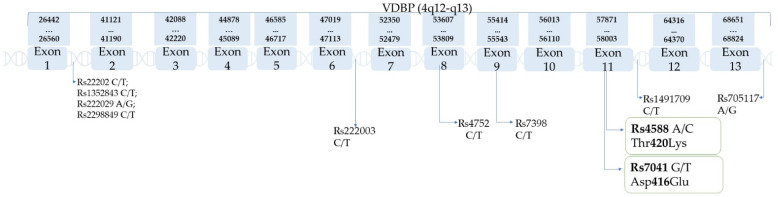
VDBP gene and its polymorphisms (based on the SNPedia database [21]).

The first step is the conversion of vitamin D to 25OHD in the liver by 25-hydroxylase enzymes. Enzymes with this activity are identified as: CYP3A4, CYP2R1, CYP27A1, CYP2J1, CYP2C11, CYP2D25 [19]. The production of the hormonally active form: 1α,24-dixydroxyvitamin D (1α,25(OH)_2_D3) is catalyzed by CYP27B1: 25-hydroxyvitamin D-1 α-hydroxylase. An active form acts with the vitamin D receptor (VDR) [19]. There is also an alternative pathway that starts with the action of CYP11A1 on D3 and produces 20(OH)D3, 22(OH)D3, 20,23(OH)2D3, 20,22(OH)_2_D3, and 17,20,23(OH)_3_D3. Hydroxylation of some of these metabolites can occur through the activity of CYP27B1 at C1α, by CYP24A1 at C24 and C25, and by CYP27A1 at C25 and C26 [16,20]. Possible pathways are shown in Figure 2 based on SNPedia base [21]. Mitochondrial CYP24A1 catalyzes the first step of 25(OH)D3 and 1,25(OH)_2_D3 degradation by 24- or 23-hydroxylation [22]. CYP11A1 was also found to be expressed in extrarenal and extragonadal tissues [23] and also in the immune system [17]. CYP11A1 vitamin D metabolites are also detectable in the serum [16]. 

Vitamin D receptor is a member of the nuclear receptor superfamily and plays a crucial role in the actions of vitamin D [24]. VDR mediates many genomic and non-genomic effects of vitamin D. Many biological pathways and networks are influenced by VDR, for example metabolism [25], including bone metabolism and remodeling [26,27], immunity and immune response [24,28], cell proliferation and differentiation [24], and cell health [29]. VDR regulates the expression of numerous genes and is involved in calcium/phosphate homeostasis [24]. Many genes are up-regulated (CYP24A1, osteocalcin or *RankI*) or down-regulated (parathormone—PTH, CYP27B1) due to VDR activation [24]. Slominski et al. reported the alternatives to VDR nuclear receptors that are involved in vitamin D metabolite signaling pathways: retinoic acid-related orphan receptors (RORα–γ; NR1F1–3) [30], AhR [31], and LXR [32].

Vitamin D binding protein was initially named the ‘group-specific component’ (Gc) by Hirschfield in 1959 after isolation from the α2-globulin portion of plasma [33]. As a result of the binding and transport of vitamin D analogs, the DBP name was adopted. After discovering macrophage-stimulating activities, VDBP was renamed as the macrophage-activating factor (GcMAF/DBP-MAF) [34]. The name has been changed several times, as many different biological functions of VDBP have been discovered [34,35]. VDBP binds to fatty acids and actin monomers and also has immune functions independent of vitamin D transport [35], such as binding to leukocyte membrane proteoglycans and the activation of the complement C5 system [18]. VDBP is well known for its single nucleotide polymorphisms (SNP), and the most common are rs7041 and rs4588, located in exon 11 of the VDBP gene [18,36]. SNPs are the most abundant genetic variants in genomes [37]. SNPs may affect protein stability, folding, flexibility, and aggregation; functional sites, reaction kinetics, and dependence on environmental parameters, such as pH, salt concentration, and temperature; protein expression and subcellular localization; and protein–small molecule, protein–protein, protein–DNA, and protein–membrane interactions [38]. Many studies have shown associations between SNPs and the concentration of protein as well as substance protein transport via VDBP in this particular case [18,39,40]. The effects of vitamin D supplementation according to the most common polymorphisms of the vitamin D binding protein was studied by Al-Daghri et al., and it was shown that 25[OH]D concentrations were significantly higher among people with the major homozygous rs7041 genotype. Post supplementation 25[OH]D was higher in participants carrying homozygous major genotypes in rs4588 and rs7041 compared to other genotypes [41].

The vitamin D binding protein has a single binding site for all vitamin D metabolites and has a high affinity for 25OHD and 1,25(OH)2D [42], but has no affinity for lumisterol and minimum affinity to tachysterol [13].

Possible haplotypes are as follows: Gc1f(1f): rs7041(T) + rs4588(C); Gc1s(1s): rs7041(G) + rs4588(C); Gc2(2): rs7041(T) + rs4588(A). According to some publications, in which rs2282679 is used as a proxy for rs4588, rs2282679(A) is typically coinherited with rs4588(C) and vice versa. Since human have two copies of each gene, it leads to six possible VDBP phenotypes [21] presented in Table 1. Table 2 present SNPs of VDBP, and chromosome location. Table 3 present the frequency of rs7041 and rs4588 among populations and geographic regions. Figure 2 show the location of the most common SNPs of the VDBP gene, and their loci in exons and introns are pointed out with arrows. 

Our review focuses on the most important correlations between VDBP polymorphisms and selected diseases described in the latest scientific reports garding obesity, polycystic ovary syndrome, metabolic syndrome, diabetes mellitus, asthma, pulmonary tuberculosis, chronic obstructive pulmonary disease, coronary artery disease, multiple sclerosis, and Parkinson’s Disease. This study is supplementation of our previous review concerning the role of VDBP on malignant tumors [18].

## 2. Diseases

### 2.1. Bone Density

Osteoporosis is a skeletal disease that affects women older than 50 years of age. During the past few years, it has been a serious public health problem because of the high socioeconomic burden. Patients suffer from deterioration of bone microarchitecture, low bone mineral density (BMD), and increased risk of fragility fractures [45]. The first studies on the effect of vitamin D supplementation on bone density showed that vitamin D (with calcium) reduced bone loss measured in the femoral neck, spine, and total body during the 3-year study and reduced the incidence of non-vertebral fractures [46]. The study by Martinez-Aguilar et al. supports the correlation of low serum VDBP levels with low BMD (osteopenic and osteoporotic). VDBP could be considered a novel, potential, and non-invasive biomarker for the early detection of osteoporosis [45]. The study by Rivera-Paredez et al. supports the association of VDBP and bone health. The article showed that the rs7041 G allele is associated with a higher level of VDBP and BMD compared to homozygous TT. The A allele of rs4588 was associated with a lower VDBP and BMD compared to homozygous CC. Among men, no association was found between these polymorphisms and VDBP, but GC variants were associated with VDBP levels. In both the women and men subgroup, no association was observed between free and bioavailable 25(OH)D and BMD [47]. Among women and adolescents, the GC genotype was associated with susceptibility to low 25(OH)D levels. The study included 198 healthy girls aged 10–18 years. The AA genotype of rs4588, TT genotype of rs7041, and CT-AT/AT-AT (GC 1f-2/2-2) genotypes were significantly associated with lower 25(OH)D levels, even after adjustment for age and season at the time of blood collection [48]. Studies of Lauridsen et al. also support the VDBP role in premenopausal bone fracture risk among white women aged 45–58, as Gc2-2 is considered to increase the risk of bone fracture compared to Gc1-1 [49]. The results of Ezura et al. indicated a complex combined effect of VDBP SNPs that underlie susceptibility to low BMD and osteoporosis. The genotyping of 13 SNPs among 384 participants and the analysis of results showed that not only a single SNP, but also a combination of them could act as a risk factor of osteoporosis. Five SNPs (39C > T, IVS1 + 827C > T, IVS1 + 1916C > T, IVS1 − 1154A > G, and IVS11 + 1097G > C) had a significant correlation with radial BMD, and IVS11 + 1097G > C located in intron 11 was the most correlated [50].

### 2.2. Obesity

Obesity contributes to reduced life expectancy, poor quality of life, cardiovascular diseases, type 2 diabetes, osteoarthritis, and cancer [51]. Serum vitamin D was found to be lower in obese people [52]. Obesity increases the risk of vitamin D deficiency among different population groups. Higher body mass index (BMI), waist circumference, and the sum of skinfolds were statistically significantly associated with lower 25[OH]D levels and with higher levels of PTH [53]. According to genetic studies, higher adiposity also causes an increased concentration of 25-hydroxyvitamin D, which is used as a vitamin D status indicator [54]. Another study showed that among the haplotypes rs7041 and rs4588, GC2-2 (rs7041 AA and rs4588 TT) has the lowest 25[OH]D levels compared to other haplotypes that contained at least one copy of the Gc1 allele (*p* < 0.0001) [55]. Interestingly, it was also observed that VDBP gene rs7041 polymorphism might be associated with the risk of obesity. In obese patients, a difference was found in the gene type TG + GG and TT frequency of rs7041 between obesity and control groups (*p* = 0.020). The G allele frequency was higher compared to the control group (*p* = 0.023). The TG and TG + GG of VDBP gene rs7041 polymorphism increased the risk of obesity after including age and gender [39]. The study of Almesri et al. showed that rs7041-G and the rare GG genotype were associated with an increase in BMI (*p* = 0.007 and *p* = 0.012, respectively) and had no influence on 25OHD3 levels. On the other hand, rs2282679 (A) and rs4588 (C) were associated with low 25[OH]D3 plasma levels (*p* = 0.039 and *p* = 0.021, respectively). There was no association between rs2282679 (A), rs4588 (C), and BMI in general, but after categorizing patients into subgroups based on their sex, it was shown that rs7041 GG was associated with high BMI in females (*p* = 0.003), and rs4588 CC was associated with high BMI in females (*p* = 0.034) and low levels of 25OHD3 in males (*p* = 0.009). Furthermore, rs12721377 AA was associated with low 25[OH]D3 levels in females (*p* = 0.039) [56].

### 2.3. Polycystic Ovarian Syndrome (PCOS) and Metabolic Syndrome (MetS)

All participants from Santos et al. were genotyped for polymorphisms rs2282679, rs4588, and rs7041, and serum 25(OH)D levels were determined. Women with PCOS were at a younger age and had significantly higher body mass index, blood pressure, and insulin resistance than the control group (*p* < 0.05). The 25(OH)D levels were lower among PCOS women with MetS, but no association was observed between PCOS and polymorphisms of VDBP. Above that, PCOS participants with MetS had a higher frequency of the TT genotype in rs7041 [57]. The study by Haldar et al. did not show significant differences in the frequency of rs7041, rs4588, and rs2060793 genotypes in PCOS and control women. The GT allele of rs7041, as well as the allelic combination of Gc1F/1F (T allele of rs4588 and C allele of rs7041; *p* value = 0.03), were associated with an increased risk of developing PCOS in vitamin D deficient women [36].

### 2.4. Postmenopausal Women

Postmenopausal women can exhibit biochemical signs of vitamin D insufficiency. Vitamin D is related to bone integrity, and 25-hydroxyvitamin D is a reliable clinical indicator of vitamin D status. Low levels of vitamin D have been linked to secondary hyperparathyroidism, increased bone turnover, reduced BMD, and increased risk of osteoporotic fractures [58]. As VDBP plays a crucial role in vitamin D transport, the study of Pop et al. showed that lower estradiol levels are associated with lowering VDBP levels [59]. Studies by Sinotte et al. showed that 25(OH)D concentrations in premenopausal women are strongly associated with higher VDBP polymorphisms. Rs7041 and rs4588 were associated with lower 25[OH]D concentrations. Rare alleles of rs7041 (TT genotype) and rs4588 (AA genotype) were associated with the lowest levels of vitamin D3 in a period of low (November to April) and high (May to October) vitamin D load [60]. The study by Alharazy et al. shows that rs7041 among postmenopausal women in Saudi Arabia was associated with total 25(OH)D, and rs4588 did not show an association with total or free levels of 25(OH)D [40]. The results presented by Santos et al. suggest that rs2282679 and the DBP GC2 isoform are related to lower serum levels of DBP and with susceptibility to 25(OH)D deficiency in adults and postmenopausal women [57]. Lauridsen et al. showed that the DBP-phenotype is linked with premenopausal bone fracture risk in perimenopausal white women (595 subjects, age 45–58). There was a significant difference in bone fracture risk among women with different DBP-phenotypes (relative risk of 0.32 in Gc2-2, compared with Gc1-1) [49].

### 2.5. Diabetes Mellitus (DB)

Diabetes mellitus is a group of dysfunctions as a result of hyperglycemia characterized by insulin resistance (IR) and secretion or excessive secretion of glucagon. Of the two types of diabetes, type 2 (T2D) is more common and connects a problem of impaired glucose regulation with a combination of dysfunctional pancreatic beta cells and insulin resistance. However, the main risk is obesity (where abdominal is the highest risk of all types). Type 1 (T1D) is an autoimmune disorder that leads to the destruction of pancreatic beta-cells [61].

#### 2.5.1. Diabetes Type 1 (T1D)

The meta-analysis of the five studies presented showed no association between rs7041 and rs4588 polymorphisms with the risk of T1D [62]. 

#### 2.5.2. Diabetes Type 2 (T2DM)

The study by Zhao et al. showed that there was a significant multiplicative interaction between rs7041 and body mass index (BMI) associated with elevated blood glucose levels and a higher BMI (>28.47), and the carrying allele G was given a stronger effect than the genotype of TT. In conclusion, the interactions between GC rs7041–CYP2R1 (enzyme in the vitamin D metabolic pathway), rs1993116, and GC rs7041-BMI may explain the mechanisms by which these may increase the risk of developing T2DM [63]. Another study by Fawzy et al. examined the frequency distribution of GC-rs7041 and showed no difference between patients and healthy controls, while GC-rs4588 showed an association with T2DM in all genetic models. The rs4588 AA variant was correlated with higher serum GC globulin, albuminuria, and poor glycemic control. On the other hand, a higher frequency of rs7041*TT and rs4588*AA was noticed in the macroalbuminuria vs. normoalbuminuric group. Patients with the GC-2 haplotype were approximately 2.5 times more likely to develop diabetes and had higher levels of albuminuria [64]. Other results were obtained in the experiment by Klahold et al. during a case-control cohort study that was conducted to investigate an association of SNPs in the vitamin D metabolic pathway with T2D. Up to 464 T2D patients and 292 healthy controls were genotyped. Patients with genotypes CYP27B1 rs10877012 “CC” (pc = 4 × 10^−5^), VDBP rs7041 “GG” (pc = 0.003), rs4588 “CC” (pc = 3 × 10^−4^), CYP24A1 rs2585426 “CG” (pc = 0.006), and rs2248137 “CG” (pc = 0.001) showed lower 25(OH)D3 and VDBP rs4588 “CC” lower 1,25(OH)2D3 levels (pc = 0.005). This study supports that vitamin D deficiency is highly prevalent in type 2 diabetes and most patients are also functionally affected by low levels of the active metabolite 1,25(OH)2D3. Furthermore, vitamin D system genes can affect the risk of type 2 diabetes and 25(OH)D3 concentration when compared to the healthy group. Despite this, the underlying mechanism has not been clarified, and trials, as well as functional studies, appear to be necessary to identify mechanisms by which the vitamin D system affects the pathophysiology of T2DM [65].

### 2.6. Asthma

Asthma is a chronic disease affecting inflammation in the lungs and airways. Common symptoms of the disease are a cough, chest tightness, and shortness of breath. Inflammation causes an overabundance of eosinophils, mast cells, activated T helper lymphocytes, and aids in identifying inflammatory mediators [66,67]. Vitamin D has been studied in asthma progression. Levels of vitamin D were significantly decreased in asthmatic patients in comparison to control patients. Results of genotyping the rs7041 among Kurdish patients showed that the GG genotype, as well as VDBP levels, was increased among the asthmatic group compared to the healthy controls (*p* = 0.003). Asthma progression was increased among patients carrying the rs7041 GG genotype [68]. A study by Fawzy et al. on s group of Egyptian patients showed that the rs7041 GG genotype is correlated significantly with asthma disease, while rs4588CA and AA genotypes were found as protective [69]. Another study supported the importance of rs7041 with the GC1S haplotype, especially among children diagnosed with asthma. The GC1S haplotype was considered to increase the risk of respiratory syncytial virus bronchiolitis in childhood and subsequent asthma development. The GC1s haplotype is associated with higher VDBP levels, which results in less free available vitamin D [70]. A study by Paraskakis et al. on a group of children with asthma showed a higher frequency of the rs7041 G allele and the A allele in rs4588 as a lower frequency allele in children with controlled asthma [71].

### 2.7. Pulmonary Tuberculosis (PTB, TB)

Tuberculosis is most often acquired through mycobacterial inhalation. It starts as an infection focus in the lung parenchyma (primary tuberculosis). It begins with necrotizing bronchopneumonia and progresses rapidly to necrotizing granuloma. Mycobacteria from the lungs spread only through the lymphatic system to the lymph nodes of the hilum drainage, where they cause necrotizing granulomatous inflammation [72]. As innate immunity plays an important role in the pathophysiology of tuberculosis, vitamin D with its transporter protein VDBP and its nuclear receptor vitamin D receptor can play a potential role in altering host defense against Mycobacterium tuberculosis. Decreased serum levels of vitamin D were observed in active TB patients as compared to healthy controls (*p* < 0.001) 209 [73]. The study by Zhang et al. showed [74] two less common VDBP polymorphisms, rs3733359 GA and rs16847024 CT, that were significantly associated with a reduced risk of PTB, as well as alleles rs3733359 A and rs16847024 T that were associated with a decreased susceptibility to PTB. In one of the most common polymorphisms, rs4588, the GT genotype was significantly higher in patients with PTB when compared to controls. The findings of Harishankar et al. [75] support those mentioned above, as the CA genotype rs4588 was associated with susceptibility to TB [OR: 1.47 (0.85–2.55); *p* = 0.049] and associated with 47.4% deficiency of 25(OH)D in patients with PTB, but the AA genotype was significantly associated with protection from TB [OR: 0.14 (0.02–1.29); *p* = 0.042]. No association was found with rs7041 polymorphism. Gene variants with 25(OH)D deficiency did not reveal a significant association due to the limited sample size, but the results showed a tendency towards 25(OH)D deficiency in rs7041 TG and rs4588 CA [75].

### 2.8. Chronic Obstructive Pulmonary Disease

Vitamin D deficiency was associated with increased risks of chronic obstructive pulmonary disease (COPD). However, the mechanism remains unknown. The vitamin D metabolite 1,25(OH)2D3 reinforced physical interactions between the vitamin D receptor with NF-κB p65 and c-Jun. The results of Fu et al. show that vitamin D is inversely correlated with inflammatory signaling in patients with COPD, and vitamin D may be a vital mediator of the progress of COPD in patients with low vitamin D levels [76]. The study of Li et al. also shows that COPD patients are at high risk of vitamin D deficiency, and the severity of COPD is inversely correlated with vitamin D levels. Furthermore, the homozygous carrier of the rs7041 T allele influences serum levels of 25OHD and is related to the susceptibility of COPD, which could be a potential candidate gene for screening COPD [77]. Among COPD smokers, high frequencies of rs7041/rs4588 haplotypes were homozygous GC1S/1S (42.5%), and higher levels of VDBP in the sputum were observed in stage I and II of COPD, only in the genotype GC1S/1S compared to non-smokers (*p* = 0.034 and *p* = 0.002, respectively) [78]. The studies of Horita et al. included 1712 patients and 1181 non COPD controls among Asians and Caucasians. The prevalence of each allele among the control group was: GC-1F 14.0%, GC-1S 53.8%, and GC-2 31.9%. Compared to GC-1S, the GC-1F allele and the GC-2 allele were associated with the risk of COPD with pooled odds ratios of 1.44 (95% CI 1.14–1.83, *p* = 0.002) and 0.83 (95% CI 0.69–0.996, *p* = 0.045), respectively. In comparison to the 1S-1S genotype, the 1F-1F genotype was a risk factor of COPD with a pooled odds ratio of 2.64 (95% CI 1.29–5.39, *p* = 0.008). The VDBP GC-1F allele was a risk for COPD in the recessive model [79]. Another study showed that patients carrying C allele at rs4588 exhibited a higher frequency of exacerbations (*p* = 0.0048), and a greater susceptibility to chronic obstructive pulmonary disease (*p* = 0.0003), as well as emphysema (*p* = 0.0029), and a tendency for rapid decline of airflow obstruction (*p* = 0.0927) [80]. The meta-analysis of Khanna et al. proves that VDBP is a major determinant of vitamin-D metabolism and transport, showing that alleles GC1F and GC1F/1F posed a risk of COPD. GC1S-1S was found to be a risk only among European participants in these studies [81].

### 2.9. Coronary Artery Disease

Cardiovascular diseases (CVDs) are the leading cause of death worldwide, and among CVD coronary artery disease (CAD), they are almost half of all cardiovascular deaths and the most common cause of death [82]. VDBP and its genetic polymorphisms have been linked as susceptible components for CAD [83]. The study by Peri et al. showed evidence of the association of rs4588 and rs7051 with CAD cases among patients after acute myocardial infarction and correlations of these polymorphisms with serum levels of 25-hydroxyvitamin D 25(OH)D. Rs4588 T/T was determined as a susceptibility factor for anteroseptal myocardial infarction, where the same genotype was generally more prevalent in smokers [82]. A study by Tarighi et al. among the Iranian population showed a significant association between the GG genotype (rs7041) and CAD (*p* = 0.02, OR = 0.5737 95% CI = 0.304–0.944) [83]. Daffara et al., in a group of 1080 patients, proved that 57% carried the mutated G allele of rs7041, while 22% carried the A allele of rs4588. In addition, higher C-reactive protein levels were observed in the carriers of the G allele of rs7041 (*p* = 0.02), and 25-hydroxyvitamin D levels were similar between the groups. The rs4588 A allele was associated with a higher prevalence of lesions in the left anterior descending artery and a longer lesion length (*p* = 0.04 and *p* = 0.03, respectively). Rs7041 and rs4588 did not affect the prevalence of CAD [84].

### 2.10. Multiple Sclerosis (MS)

Numerous studies suggest that vitamin D levels affect the risk of multiple sclerosis development and modify disease activity [85,86,87,88,89]. Munger et al. found an inverse relationship between serum 25(OH)D level and risk of MS among Caucasians. No similar association was found among those with darker skin or Hispanics [88]. According to the research, these groups have lower 25-hydroxyvitamin D levels with no significant health consequences [90,91]. There are racial/ethnical differences in the polymorphism of the vitamin D-binding protein-SNPs at rs7041 and rs4588. The dominant allele in rs7041 in Caucasians is the minor allele in those with African heritage. The VDBP isoform most commonly found in individuals with darker skin is the most efficient transporter of 25-hydroxyvitamin D and its metabolites. The MS Sunshine study found that there is a strong association between higher lifetime sun exposure and MS risk across racial/ethnic groups. Low ultraviolet radiation from the sun should lead to low vitamin D status and can explain the geographic distribution of the disease. There is a lack of association between 25-hydroxyvitamin D and MS risk among those with African heritage and Hispanics [92]. Langer-Gould et al. suggested that these differences cannot be explained by racial/ethnic variations in bioavailable vitamin D [92]. Xin Zhang et al. provide evidence that VDBP rs7041 and 4588 polymorphism may not be associated with an increased risk of multiple sclerosis in the meta-analysis [62].

### 2.11. Parkinson’s Disease

Low vitamin D status is suggested to be associated with Parkinson’s Disease [93,94,95]. Knekt et al. found that individuals with a serum vitamin D concentration of at least 50 nmol/L had a 65% lower risk than those with values below 25 nmol/L, after the adjustment of several potential cofounders, in the follow-up for Parkinson’s disease occurrence of the Mini-Finland Health survey [96]. Zheng Lv et al. suggested that patients with 25(OH)D level <50 nmol/L experienced a twofold increased risk of PD in the meta-analysis [97]. In the prospective cohort study of 137 patients with Parkinson’s disease, circulating 25-hydroxivitamin D levels were deficient in one-half of the patients [98]. GC polymorphism was associated with 25(OH)D levels-TT carriers for GC1, and AA carriers for GC2 had lower vitamin D status. There was no significant association between GC polymorphism and 1,25(OH)D levels. SNPs of VDBP showed no significant association with the severity of PD. In another study of 382 patients and 242 controls in a Turkish cohort, only rs7041 was associated with PD risk [94]. Significantly higher levels of serum 25-hydroxyvitamin D was observed in the group of homozygous major allele carriers for rs2282679, rs3755967, and rs2298850 with slower progression of the disease. In the proteomic studies, a decreased level of VDBP in the CSF has been suggested to be a biomarker of disease [99].

All the information above in the Section 2 is gathered into summary table—Table 4.

## 3. Research Limitations on Vitamin D

Vitamin D and its metabolites have some measurement limitations. Even if both serum and plasma can be used for vitamin D metabolite measurements, serum is preferred due to the fact that it is free from anticoagulants (heparin, EDTA, citrate). EDTA, heparin, and citrate may interfere with measurements [100]. The second problem in measuring is the stability of vitamin D metabolites because metabolites are only stable due to the binding to VDBP and proper storing: room temperature, 4 °C, or frozen. In case of separation from VDBP, storing at −70 °C is required [101]. Seasonal variation of vitamin D also needs to be considered as vitamin D biosynthesis is sun-dependent and depends on geographical locations and seasons (highest levels of vitamin D during the summer, lowest during the winter) [102]. This information may provide many significant differences in studies concerning vitamin D and its metabolites and its connection to polymorphisms occurring in the vitamin D pathway. Genetic variants of VDBP [103], DHCR7 [103], CYP2R1 [104], CYP24A1 [104], VDR [104], CYP3A4 [105], CYP2R1 [105], CYP27B [105], and LRP2 [105] were found to be associated with 25(OH)D levels. There are still many polymorphisms that have not yet been included in any studies.

Other important factors are age, sex, BMI, and lifestyle. Age can play a role, mostly in the group of elderly people >75 years old, due to reduced calcium absorption, intestinal resistance of calcium absorption to circulating 1,25(OH)_2_D, decreased ability of skin in vitamin D producing, deficiency of vitamin D substrate [106], and less sunshine exposure [107]. BMI is also considered to increase with age. Vitamin D deficiency is prevalent in a group of obese people which suggests the correlation of higher levels of adipose tissue and lower levels of vitamin D status [108]. Skin color also plays a role in vitamin D levels, as darker skin tones protect from UVB irradiation and, consequently, increase the risk of vitamin D deficiency. In addition, darker-skinned individuals have slower vitamin D synthesis [100]. 

The liver and kidneys are the two most important organs in the metabolism of vitamin D. Decreased kidney and liver functions may provide calcitriol deficiency and disruptions in overall vitamin D catabolism [100]. Diseases associated with these organs and their relationship with vitamin D and its metabolic pathway still require more research.

We suggest that all experiments that include vitamin D and its metabolism should contain more specific information concerning the studied group, age, sex, and geographical location. Articles without such information may lead to misleading conclusions. 

## 4. Conclusions

Vitamin D and vitamin D binding protein have an undeniable impact on human health. Polymorphisms occurring in the VDBP gene can be a significant risk factor or prevalence factor in many diseases associated with obesity, diabetes, PCOS, MD, or PD. Many studies have shown VDBP and vitamin D level as a biomarker in many diseases, and if that is so, knowing the role of SNPs in proteins may contribute to finding new approaches for many syndromes and diseases associated with the vitamin D metabolic pathway.

In bone density, low levels of VDBP were associated with lowering BMD in the rs7041 “G” allele., while rs4588 “A” was linked to lower 25(OH)D levels and increased bone fracture risk. In obesity, the rs4588 “C” allele was linked to lower 25(OH)D levels and higher BMI scores among females. Rs4588 “T” and rs7041 “C” alleles increased risk in developing PCOS among women who had vitamin D deficiency. In COPD, the rs7041 “T” allele was associated with a higher risk of disease. 

We believe that VDBP polymorphisms may affect the levels of vitamin D metabolites and thus contribute to the development of certain diseases. However, we are aware that most studies do not have a specific time of the year to measure vitamin D accurate metabolite levels, which can be misleading due to its seasonal nature. Research to date, although linking some diseases with vitamin D deficiency or VDBP polymorphisms, is not sufficient if the underlying mechanism is not elucidated. For this purpose, further research is required regarding the possible influence of SNP polymorphisms, their modifications in the structure of the binding protein, and their influence on the organism.

## Figures and Tables

**Table 1:** Characteristics of vitamin D binding protein polymorphisms in most common variants (based on Rozmus et al. 2020 with modifications [18,21]).

Variant	Version
GC1S/1S	rs7041(G;G)
GC1S/1F	rs7041(G;T) AND rs4588(C;C), or, rs7041(G;T) AND rs2282679(A;A)
GC1S/2	rs7041(G;T) AND rs4588(A;C), or, rs7041(G;T) AND rs2282679(A;C)
GC1F/1F	rs7041(T;T) AND rs4588(C;C), or, rs7041(T;T) AND rs2282679(A;A)
GC1F/2	rs7041(T;T) AND rs4588(A;C), or, rs7041(T;T) AND rs2282679(A;C)
GC2/2	rs4588(A;A) or rs2282679(C;C)

**Table 2:** SNPs alleles and chromosome location (based on SNPedia [21]).

SNP	Location	Major Allele	Minor Allele
rs7041	exon 11	G	T
rs4588	exon 11	C	A
rs1155563	intron 1	T	C
rs1352844	intron 1	C	T
rs1352845	intron 1	A	G
rs222016	intron 2	A	G
rs2282679	intron 11	A	C
rs705119	intron 11	C	A
rs12512631	3′ downstream	T	C
rs222049	3′ downstream	C	G
rs3733359	5′ UTR	G	A

**Table 3:** Frequencies of alleles in rs4588 and rs7041 among different populations and geographic regions based on [43,44].

Geographic Region/Population	Sample Size (*n*)		Allele Frequencies	Allele Frequencies	References
	rs4588 *	rs7041 **	rs4588-T	rs7041-A	
Estonian	4480	4480	0.3036	0.4125	[41,42]
Korean	2930	nd. ***	0.2843	nd. ***	
Northern Sweden	600	600	0.242	0.375	
Daghestan	1136	1134	0.2764	0.4462	
Vietnamese	614	nd.	0.22	nd. ***	
Finland	304	304	0.188	0.355	
Quatari	216	216	0.199	0.486	
Siberian	nd. ***	34	nd. ***	0.26	
European	263394	285118	0.281206	0.433	
African	10488	11716	0.09392	0.8182	
African American	10118	11306	0.09567	0.81523	
Asian	6536	6908	0.2852	0.7351	
East Asian	4624	4946	0.2885	0.7351	
Other Asian	1912	1962	0.2772	0.7243	
Latin American individuals with Afro-Caribbean ancestry	1252	1488	0.2236	0.541	
Latin American individuals with mostly European and Native American Ancestry	2188	7238	0.1846	0.4823	
South Asian	314	5226	0.226	0.4351	
Other	21820	18956	0.26801	0.49625	

* [42]. ** [43]. *** nd means no data.

**Table 4:** Summary of mentioned diseases in the Section 2. Most common VDBP polymorphisms with effects.

	Polymorphisms	Effects	Group Characteristics	References
Bone density	rs7041 “G”	Low VDBP = low BMD;	women, aged ≥ 45 years old, 446 participants	[45]
		Higher VDBP and higher BMDlevels;	1853 adults, aged ≥ 18	[47]
	rs7041 “T”	Lower 25(OH)D levels;	198 girls, aged 10–18 years old	[48]
	rs4588 “A”	Lower VDBP and lower BMD levels;	1853 adults, aged ≥18	[47]
		Lower 25(OH)D levels;	198 girls, aged 10–18 years old	[48]
		Increasing bone fracture risk;	595 women	[49]
Obesity	rs7041 “G”	G and GG associated with higher BMI in females; low 25OHD in males	406 adults	[56]
		Increasing BMI; no effect on 25(OH)D levels;		
	rs2282679 “A”	Lower 25[OH]D3		
	rs4588 “C”			
		High BMI in females		
	rs12721377 “A”	Low 25[OH]D3 levels in females		
PCOS	rs7041 “T”	PCOS + metabolic syndrome: significantly higher body mass index, blood pressure, and insulin resistance	443 healthy women aged 20–62 years, 359 of them were postmenopausal	[57]
	rs4588 “T” and rs7041 “C”	Increased risk of developing PCOS in vitamin D deficient women	100 women, 50 healthy and 50 with PCOS	[36]
Diabetes mellitus T2	rs7041 “G”	Elevated blood glucose levels; higher BMI	2271 adults	[63]
	rs7041 “G”	lower 25(OH)D3 and VDBP levels	553 patients, 916 controls	[65]
	rs4588 “C”			
	rs4588 “CC”	lower 1,25(OH)2D3 levels		
	rs4588 “A”	Higher serum GC globulin, albuminuria, and poor glycemic control (Patients more likely to develop diabetes)	200 participants. 120 with DMT2, 80 controls	[64]
Asthma	rs7041 “G”	Increasing VDBP levels; increasing asthma progression	110 patients with asthma, 110 healthy controls	[68]
		Correlated significantly with asthma	192 children and adolescents (96 with asthma and 96 healthy controls)	[69]
		Increasing the risk of respiratory syncytial virus bronchiolitis in infancy and subsequent asthma development	198 healthy children with families	[70]
	rs4588 CA and AA	Protective effect	192 children and adolescents (96 with asthma and 96 healthy controls)	[69]
Tuberculosis	rs3733359 “A”	Decreased susceptibility to PTB	490 PTB cases and 489 healthy controls)	[74]
	rs16847024 “T”			
	rs4588 CA	Associated with susceptibility to TB	125 PTB cases and 125 healthy controls	[75]
		Associated with 47.4% deficiency of 25(OH)D in patients with PTB		
	rs4588 CA	Protective effect		
COPD	rs7041 “T”	Related to susceptibility of COPD	250 participants: 116 COPD patients with smoking history and 134 healthy smokers	[77]
		Associated with the risk of COPD	1712 subjects: 531 COPD cases and 1181 controls.	[79]
	Rs4588 “C”	Susceptibility to COPD, emphysema	361 COPD patients and 219 control	[80]
CAD	rs7041 “G”	Significant association with CAD	143 men with CAD and 145 healthy	[83]
	Rs4588 “A”	Higher prevalence of lesions in the left anterior descending artery and a longer lesion length	1080 patients	[84]
PD	rs7041, rs4588	No significant association with the severity of disease	137 patients	[98]
	rs7041	Rs7041 associated with PD risk (*p* < 0.05)	*N* = 382 PD patients and 242 healthy controls in a Turkish cohort	[94]
	rs2282679	higher levels of serum 25-hydroxyvitamin D in slower progression of disease		
	rs3755967			
	rs2298850			
MS	Rs7041 Rs4588	No significant association of polymorphism with the risk of MS	Meta-analysis of six studies	[62]
